# Supplementary material for: Comparative efficacy of schroth and core training for early postoperative recovery in adolescent idiopathic scoliosis: A single blind randomized controlled trial
Source: PLoS One. 2026 Jan 7;21(1):e0340585. doi: 10.1371/journal.pone.0340585 (PMC12779134; doi:10.1371/journal.pone.0340585)
Supplement: Attachment — Submitted filename: Response to Reviewers.docx [file pone.0340585.s001.docx]

**Response to Reviewers**

**Manuscript ID: PONE-D-25-51249**

Dear Editor and Reviewers,

We would like to sincerely thank you for your time, effort, and thoughtful

comments on our manuscript. We greatly appreciate the opportunity to revise

our work and have carefully considered all feedback provided.

In response, we have revised the manuscript accordingly to address each

comment and suggestion. Below, we provide a detailed point-by-point response to all reviewers’ comments. For each point, we first present the original comment

(in bold), followed by our response (in normal) and a description of the changes made in the revised manuscript.

We hope that the revisions we have made will meet your expectations, and we

remain grateful for your constructive guidance in improving our work.

**Section 1. Response to Editor**

Dear Dr. Mohsenifar,

Thank you for your decision and the opportunity to revise our manuscript. We are deeply grateful to you and the reviewers for your careful evaluation, constructive feedback, and insightful suggestions. We truly appreciate the time and effort invested in reviewing our work.

We have taken all comments very seriously and have revised the manuscript

accordingly. Each point raised has been addressed in detail below, and corresponding changes have been made in the manuscript to improve its clarity, rigor, and overall quality. We hope that our responses and revisions meet your expectations. We have also ensured that the revised manuscript adheres fully to the PLOS ONE style and formatting guidelines, as per the provided templates.

Funding: The funding statement was previously provided in the cover letter. The declaration was: "The funders had no role in the study design, data collection and analysis, decision to publish, or preparation of the manuscript." The information was kept out of the main text.

ORCID: I have successfully completed and linked ORCID ID to profile in the submission system. ORCID ID：0009-0004-1146-3833

Data：We have revised the Data Availability Statement. The minimal dataset required to replicate the findings of this study has been deposited in Figshare and is available at [https://doi.org/10.6084/m9.figshare.30836405](https://doi.org/10.6084/m9.figshare.30836405)

Below we provide a detailed point-by-point response to all reviewers’ comments. We hope the revised manuscript now meets the expectations of both the reviewers and the journal.

**Section 2. Response to Reviewers**

**Reviewer #1:**

**Thank you for your kind invitation to review the manuscript entitled “Comparative Efficacy of Schroth and Core Training on Optimizing Trunk Balance and Early Function in Postoperative Adolescent Idiopathic Scoliosis: A Single-Blind Randomized Controlled Trial.”
In this study, the authors aimed to investigate the effectiveness of adding Schroth exercises to core stabilization exercises in patients with AIS who underwent surgical treatment.
Rehabilitation studies conducted after scoliosis surgery are quite limited in the literature. Therefore, the authors have undertaken an important study that may fill a gap in the existing literature.
My detailed comments regarding the manuscript are presented below.**

**I Title and Abstract**

**Comment 1: The title reflects the overall content of the study; however, it may be a bit long for readers. The authors may consider shortening it slightly.**

**Response:**

We are grateful to the reviewer for their valuable suggestion. In response, we have revised the manuscript title to the following: "Comparative Efficacy of Schroth and Core Training for Early Postoperative Recovery in Adolescent Idiopathic Scoliosis: A Single Blind Randomized Controlled Trial.

**Comment 2: In this section, I recommend not using “S” for scoliosis classification. Since Schroth exercises were applied, you may use the Schroth classification, or as you focused on postoperative rehabilitation, the Lenke classification would be more appropriate.**

**Response：**

We thank the reviewer for this insightful suggestion. We agree that using 'S-shaped' and 'C-shaped' was an oversimplification and inappropriate for a surgical cohort. As correctly pointed out, we have revised the text to use the Lenke classification system. Specifically, in the abstract (line 30), we have replaced the description of "S-shaped with a major thoracic curve" with "Lenke type 1" to accurately reflect the patient population.

**Comment 3：As the study involves postoperative evaluation, the SRS-30 questionnaire, which includes postoperative assessment items, could have been used. However, since the study has already been completed, this cannot be changed.**

**Response：**

We sincerely thank the reviewer for this valuable suggestion. Upon further consideration, we fully agree that the SRS-30 questionnaire, with its additional domains specifically addressing postoperative quality of life, is indeed more suitable for studies focusing on the postoperative period. This insight provides invaluable guidance for our research group's future work in rehabilitation for AIS patients. As the present study has already been completed, we have acknowledged the limitation of not having used the SRS-30 questionnaire in the dedicated section of our manuscript (Lines 514-518).

**Comment 4：Please report some p-values in the Results section as actual numerical values.**

**Response：**

We are grateful to the reviewer for bringing this to our attention. We have reported some specific statistical values and p-values in the text regarding the statistical results.

**Comment 5：Choose keywords according to MeSH terminology.**

**Response:**

We are grateful to the reviewer for this valuable feedback. In response, we have re-selected our keywords based on the MeSH thesaurus. The new keywords: Adolescent, Scoliosis, Spinal Fusion, Exercise Therapy, are all controlled vocabulary terms within MeSH, which indeed improve the discoverability of our manuscript.

**II Introduction**

**Comment 6：Pay attention to the use of references throughout the manuscript and ensure that appropriate references are cited. For example, a validation study has been cited for the definition of scoliosis (Ref 1)**

**Response：**

We are grateful to the reviewer for bringing this to our attention. We agree that the previous reference Ref 1 was not appropriate for defining scoliosis and have therefore replaced it with a more suitable citation. The updated reference is: Diarbakerli E, Savvides P, Wihlborg A, Abbott A, Bergström I, Gerdhem P. Bone health in adolescents with idiopathic scoliosis. Bone Joint J. 2020;102-B(2):268–72. All references cited in the manuscript have been carefully examined following the suggestion.

**Comment 7: Add a reference for Lines 78–79.**
**Response:**

We thank the reviewer for the suggestion. Reference [3] (2016 SOSORT guidelines) was previously cited at line 85. We have now added an additional citation of reference [3] at lines 87 as recommended.

**Comment 8: The Introduction could be shortened and simplified. I suggest deleting sentences that interrupt the logical flow.**

**Response:**

We are grateful to the reviewer for this valuable feedback. In response, we have thoroughly revised the Introduction to enhance its conciseness and logical coherence. We have deleted several sentences that were repetitive or disrupted the narrative flow, with the majority of changes concentrated in the first and third paragraphs.

**Comment 9:Add the study hypothesis at the end of this section.**

**Response:**

We are grateful to the reviewer for this insightful recommendation. In response, we have formulated and stated the primary research hypothesis, which aligns directly with our main objective. It is hypothesized that patients undergoing the combined Schroth and core training intervention will exhibit significantly better preservation of spinal alignment and postural correction at the 6-month assessment compared to those in the active control group performing core training only. (Lines 137-140).

**III Method**

**Comment 10: In the inclusion criteria, avoid using the “S-type” scoliosis classification. Instead, select a classification method appropriate to your study and describe it in the Methods section.**

**Response：**

We would like to express my sincere thanks to the reviewer for this constructive suggestion. Based on this comment, the previous description of 'S-shaped curve with a major thoracic curve' in the inclusion criteria has been replaced with the standard 'Lenke type 1' classification. We agree that the Lenke classification provides a more accurate and clinically relevant definition for patients undergoing surgical intervention.

**Comment 11：Provide references for both the core and Schroth exercises.**

**Response:**

We thank the reviewer for this valuable comment. We have now revised the manuscript to include relevant references on both Schroth therapy and core exercise to better support our statements.

**Comment 12: Clarify where the exercises were performed — were they part of a home program or supervised sessions by a physiotherapist? How many supervised sessions were conducted?**

**How many sessions were required for the children to learn the exercises?**

**Were all exercises taught by the same physiotherapist?**

**Was the rehabilitation protocol initiated on postoperative day 1?**

**How was exercise progression ensured? Please explain for the readers.**

**Response:**

We thank the reviewer for this important question. We have provided a clearer description of the intervention protocol.

First, given that all participants were school-aged children, the 6-month intervention program could not be conducted entirely in a hospital setting under direct therapist supervision. Therefore, the program consisted of a combination of therapist-supervised sessions and home-based exercises. Specifically, patients in both groups attended one weekly supervised session at the clinic with the same therapist. For the remainder of the week, they performed an additional 2-3 home-based sessions per week, which were overseen by their parents.

Second, initial training involving 4-5 sessions conducted by the same certified Schroth PT, who ensured participants and their caregivers could accurately perform the exercises at home or school.

Third, all exercise sessions were instructed by the same therapist certified in the International Schroth Scoliosis Therapy (ISST) method. This approach was taken to mitigate the potential heterogeneity in intervention effects that could arise from differences in therapeutic skill and delivery.

Fourth, reflecting our commitment to the ERAS protocol, rehabilitation began on postoperative day 1 for both study groups. A key safety protocol required the therapist to deliver all interventions in collaboration with and under the guidance of the attending orthopedic surgeon. The initial regimen included simple breathing exercises, bed-mounted exercises for the lower limbs, facilitated ambulation, and posture control exercises utilizing mirror visual feedback.

At last, this hybrid model, with its weekly clinic visits, allowed the therapist to closely monitor each patient's performance, progressively modify the exercise regimen, and ensure the principle of gradual progression was followed throughout the intervention.

We have added a detailed description of the intervention protocol to the manuscript to provide greater clarity for readers regarding the rehabilitation procedures. (Lines 185-194)

**Comment 13：If the cases were blinded to group assignment, how was informed consent obtained? Please explain.**

**Response：**

We appreciate the reviewer's comment on this critical methodological detail. To clarify, the informed consent process was completed before group assignment took place. During this process, all adolescent participants and their parents/legal guardians received comprehensive information about the study. This included an explanation that the study was comparing two different physical therapy approaches after surgery, and that they would be randomly allocated to one of them without being told which one. The key elements of both rehabilitation programs were described in general terms (e.g., "a program focusing on core muscles" vs. "a program integrating core training with specific scoliosis exercises"), without disclosing the specific hypothesis about which was expected to be superior. This method is aligned with CONSORT guidelines for single-blind trials and was approved by our institutional ethics committee, ensuring that blinding was maintained without compromising the ethical validity of the consent. I have already added this passage to Trial design and Randomization and Blinding.

**Comment 14：Add references for all assessment methods, including the Cobb method. Indicate that the 2nd, 3rd, and 4th measurements were taken from radiographs.**

**Response:**

We are grateful to the reviewer for raising these important points. We have now supplemented the manuscript with key references pertaining to pelvic balance. Additionally, as suggested, we have provided a clearer description of the radiographic measurement process by adding the following statement: "At each predefined assessment timepoint, radiographic parameters (including Cobb angles) were measured from standing radiographs using RadiAnt DICOM Viewer software." (Lines 214-216)

**Comment 15: Provide references for the EMG evaluations and for PMSI.**

**Response:**

We are grateful to the reviewer for this valuable feedback. In response, we have incorporated appropriate citations into the manuscript to substantiate the methodologies pertaining to EMG evaluations and the PMSI.

**Comment 16: As a suggestion, the SRS-30 questionnaire could have been used for postoperative evaluation.**

**Response:**

We are grateful to the reviewer for this insightful recommendation. Our team has reviewed the SRS-30 and recognizes its superior applicability for postoperative research, particularly given the supplementary items related to postoperative quality of life that are not captured by the SRS-22. This perspective is immensely helpful for guiding our subsequent investigations into post-surgical rehabilitation for AIS. Given that the current study is finalized, we will explicitly state the omission of the SRS-30 as a study limitation in the revised manuscript.

**Comment 17:** **Statistical Analysis: Since your sample size is below 50, normality could be tested using the Shapiro–Wilk test.**

**Response:**

We appreciate the valuable suggestion from the reviewer. Given that the sample size of this study is less than 50, we have supplemented the Shapiro–Wilk test to verify the normality of the data.

**IV Results**

**Comment 18：Add the distribution of curve types to Table 1. Indicate how many vertebrae were fused and whether there was a difference between groups.**

**Respone：**

We thank the reviewer for these critical suggestions regarding the baseline characteristics.

Baseline Table (Table 1): As recommended, we have added the distribution of curve types (Lenke classification) and the number of fused vertebrae to Table 1. Statistical analysis confirmed that there were no significant differences between the two groups in these parameters, reinforcing the comparability of the groups at baseline.

In accordance with the reviewer's suggestion, we employed the Lenke classification. However, due to the limited sample size, patients were categorized into the three primary lumbar modifier subtypes (Lenke 1A, 1B, and 1C), and a further subdivision based on sagittal thoracic modifiers（hypokyphosis, normal, hyperkyphosis）was not performed.

**Comment 19： Include measurement units in the tables.**

**Respone：**

We are grateful to the reviewer for their keen observation. In response, we have conducted a systematic check of all tables and have supplemented the units of measurement accordingly to ensure full clarity.

**Comment 20：Although there are many tables and assessments, it would be beneficial to highlight group differences. Comparing the changes between groups will help determine whether one group is superior to the other.**

**Response：**

We are grateful to the reviewer for this valuable feedback. In response, we have thoroughly revised the presentation of the results in our manuscript. The changes, now visible in track-change mode, are aimed at more effectively highlighting the comparative outcomes between the two intervention groups and explicitly stating where one approach proved superior.

**V Discussion**

**Comment 21：I recommend beginning this section by clearly presenting your primary finding.**

**Response：**

We are grateful to the reviewer for this valuable insight. We fully agree that the discussion should prioritize the study's primary findings. In response, we have reframed the opening of the discussion section, removing the paragraph on muscle and self-image to place greater emphasis on comparing the outcomes between the two groups.

**Comment 22：The order of presentation of the assessment methods in the Discussion should match the order used in the Methods and Results sections.**

**Response:**

We are grateful to the reviewer for this valuable feedback on improving the manuscript's flow. In response, we have meticulously reordered the paragraphs in the discussion section to precisely mirror the sequence of the methods and results. The revised structure now logically presents: first, a summary of the primary findings; second, the radiographic outcomes; third, the surface electromyography findings of the paraspinal muscles; fourth, the results of trunk muscle strength and endurance; and finally, the health-related quality of life outcomes as measured by the SRS-22.

**Comment 23：Include studies from the literature that focus on postoperative rehabilitation in AIS and discuss your results in relation to these studies.**

**Response：**

We thank the reviewer for this insightful comment. In response, we have incorporated relevant literature on postoperative rehabilitation in AIS and expanded the discussion by comparing our findings with these studies. This new content has been added to the Discussion section (Lines 424-439). We acknowledge that high-quality studies focusing specifically on PSSE in the postoperative context are currently limited. Nonetheless, our revised discussion has addressed this point within the scope of the available evidence.

**Comment 24： As a suggestion, since spinal fusion surgery was performed, hanging exercises that disturb the sagittal plane might not have been suitable.
This is because, by nature, AIS patients tend to have hypokyphosis, and in the postoperative period, sagittal plane curvatures are further affected. Hanging exercises may potentially worsen sagittal alignment. However, this is simply a suggestion for your future studies.,**

**Response：**

We sincerely thank the reviewer for this expert insight regarding Schroth exercises. We fully agree that hanging exercises require extreme caution, not only for conservative management but also, as the reviewer rightly points out, in postoperative patients. We acknowledge that patients with major thoracic curves often present with pre-existing hypokyphosis, and surgery can further impact sagittal alignment. In our intervention, the application of hanging exercises was therefore highly selective and approached with great caution. While we recognize its benefits for spinal elongation and core activation, it was strictly contraindicated for patients with thoracic hypokyphosis, who were excluded from this specific component of the training. This careful patient selection was a crucial part of our safety protocol. Second, to mitigate the potential reduction of the thoracic kyphosis caused by hanging exercises, we ensure that during hanging movements, the feet are positioned with knees bent to engage the quadriceps in eccentric contraction. This helps stabilize the pelvis and lower limbs and reduces the weight burden from body sinking, rather than allowing the body to drop freely.

Furthermore, we are grateful for the reviewer's crucial reminder to pay heightened attention to sagittal alignment in postoperative rehabilitation. To prevent any potential misinterpretation or unsafe replication by readers, which could lead to incorrect application and exacerbate issues like thoracic hypokyphosis, we have proactively removed the image depicting the hanging exercise from the manuscript.

**Reviewer #2:**

**Comment 1: This was a simple single-blinded randomized controlled trial enrolling 46 AIS patients. The analyses intended appear to be reasonable. However, although justified based on a single rather large effect size of 1.02 using the G-Power software, the sample size is actually inadequate for the number of endpoints, time assessments and interactions. The investigators have to better justify this one effect size being relevant to all the objectives (The protocol section 3.1 of the appendix is not very helpful).**

**Response:**

Thank you very much for your insightful comment regarding the sample size of our study. We fully agree that while the sample size was calculated a priori based on the primary outcome, it may indeed be insufficient to provide adequate statistical power for all secondary endpoints, multiple time points, and interaction effects examined in the study.

We would like to clarify the rationale behind our sample size calculation:

(1) Driven by the Primary Outcome: The calculation was performed using G*Power 3.1 for a repeated-measures ANOVA. The effect size (f = 0.25) was derived from pilot data on the Cobb angle—the primary imaging outcome of this trial. We chose this as the primary outcome and basis for calculation because the Cobb angle is a key and clinically established measure for assessing structural change in AIS, representing the core effect our intervention was designed to target.
(2) Consistency in Analysis: The primary outcome was planned to be analyzed using repeated-measures ANOVA, which aligns with the statistical model used for the sample size estimation.
(3) Acknowledgment Regarding Secondary Outcomes: We completely understand that a sample size determined by the primary endpoint may not provide equivalent power for all secondary outcomes (sEMG, muscle strength/endurance, SRS-22). This is an important limitation, particularly when considering multiple comparisons.

In direct response to your comment, we have explicitly addressed this limitation in the revised manuscript. The following text has been added to the “Limitations” section (Lines 510-513): “Due to the single-center nature and modest sample size, the generalizability of this study is limited. Furthermore, the limited statistical power for secondary outcomes precluded multiple-comparison corrections, rendering these results exploratory. Consequently, larger, multi-center studies are required to validate these observations.”

We believe this addition enhances the transparency and rigor of the manuscript. Once again, we sincerely appreciate your valuable feedback, which has significantly improved the quality of our work. We agree that future large-scale, multi-center studies are warranted to confirm and clarify the effects across all endpoints.

**Comment 2：If this is a repeated measures design, then in the results , one should see** **the results for each endpoint first giving the overall treatment effect, time effect and interaction effect before doing separate analyses at each of the time points. The many comparisons being made reads like an exploratory analysis.
Also the adjustment made for multiple comparisons is not evident in the results section when everything is compared to p=0.01, 0.05 or 0.001. Please be sure that all adjustments to the type I error are , in fact, valid. For example in Table 4 the p-value for flexor endurance is not significant at 0.05 as the adjusted p-value for the four tests done in this table is 0.0125.**

**Also, specifically, what is the related samples non parametric test being used for the repeated measures ANOVA, if needed? Also, in the limitation section of the paper the reader should be cautioned of the small sample size from a single institution and that the p-values should be interpreted with caution as should the generalization of the results**

**Response：**

Thank you for your detailed and insightful critique regarding the statistical analysis and presentation of our results. We appreciate the opportunity to clarify our methodology and have substantially revised the manuscript to address your specific concerns, enhancing its rigor and transparency.

(1) Presentation of Overall Effects for Repeated Measures:

The reviewer are correct in stating that results for a repeated-measures design should first present the overall effects. In the revised manuscript, we have restructured the presentation of all outcomes analyzed with two-way repeated-measures ANOVA. For each relevant endpoint, we now explicitly report the F-statistics and p-values for the overall main effects of Time and Group, as well as the Time × Group interaction, before detailing any post-hoc comparisons. This change has been applied to the text and is reflected in the revised Tables 2-4.

(2) Adjustment for Multiple Comparisons & Clarification on Analysis Strategy:

We fully agree on the critical importance of controlling Type I error and have refined our approach as follows, which is now clearly detailed in the Statistical Analysis section:

Primary Outcomes (Imaging Parameters): For analyzing these outcomes, a p-value < 0.05 was considered statistically significant. Following a significant interaction or main effect in the repeated-measures ANOVA, all post-hoc pairwise comparisons were conducted with the Bonferroni correction to adjust for multiple comparisons.

Secondary Outcomes (sEMG, Muscle Function, SRS-22): Consistent with our a priori plan and in recognition of their exploratory nature, analyses of secondary outcomes were performed without additional family-wise error rate adjustment across different outcome domains. The Bonferroni correction was applied only to post-hoc tests following a significant ANOVA result within each individual secondary outcome analysis. This approach is now explicitly stated in the manuscript to prevent misinterpretation of their confirmatory value.

Regarding Table 4 (Muscle Endurance) & Specific P-values: To ensure consistency and clarity, we have re-analyzed all data, ensuring that the reported p-values for post-hoc tests in tables and text are the Bonferroni-adjusted values where applicable. Any instance where a raw p-value was previously reported has been corrected. The interpretation of results, particularly for measures like flexor endurance, now strictly adheres to these corrected thresholds.

(3) Specification of Statistical Tests and Data Handling:

Parametric Analysis Pathway: As described in the methods, continuous variables were assessed for normality using the Shapiro-Wilk test. For data meeting the normality assumption, a standard two-way repeated-measures ANOVA was used. The assumption of sphericity was evaluated using Mauchly's test, with the Greenhouse-Geisser correction applied when violated.

Handling of Non-Normal Data: For data not normally distributed, our protocol was to apply a logarithmic transformation. If the transformed data then met the normality assumption (confirmed via S-W test), they were analyzed using the two-way repeated-measures ANOVA as described above. This rationale and sequence are now clearly outlined.

(4) Strengthened Limitations Section:

In direct response to the reviewer’s recommendation, we have significantly expanded the Limitations section. We now explicitly caution readers about:

The single-center design and modest sample size, which limit the generalizability of our findings. The resulting limited statistical power, particularly for the analyses of secondary exploratory outcomes. The need for caution in interpreting the p-values from secondary analyses, which are presented as exploratory and hypothesis-generating. The necessity for future large-scale, multi-center randomized controlled trials to confirm our results. (Lines 510-513)

We believe these comprehensive revisions, which align precisely with the detailed statistical methods now reported, have fully addressed your concerns. Thank you again for your expert feedback, which has been instrumental in improving the quality and credibility of our manuscript.

**TABLE 2.** Comparative analysis of postural optimization outcomes between the two patient cohorts.

| Outcomes |  | T1  Mean ± SD | T2  Mean ± SD | T3  Mean ± SD | *F_group_* , *P_group_* | *F**_time_* , *P_time_* | *F_(time*group)_* | *P_(time*group)_* | Pairwise  comparison |
| --- | --- | --- | --- | --- | --- | --- | --- | --- | --- |
| Major-curve Cobb (°) | SCT | 15.88±7.37 | 15.38±7.64 | 15.72±7.94 | 0.640, 0.487 | 0.026, 0.873 | 0.426 | 0.601 | None |
|  | CT | 16.55±7.64 | 16.55±7.58 | 16.38±7.87 |  |  |  |  |  |
| Minor-curve Cobb (°) | SCT | 10.27±6.01 | 12.05±9.39 | 11.94±5.68 | 0.674, 0.482 | 0.277, 0.602 | 0.416 | 0.661 | None |
|  | CT | 12.05±9.39 | 11.44±8.36 | 12.44±8.17 |  |  |  |  |  |
| Shoulder balance (cm) | SCT | 0.60±0.58 | 0.62±0.55 | 0.50±0.48 | 0.003, 0.990 | 2.732, 0.108 | 0.676 | 0.480 | None |
|  | CT | 0.78±0.74 | 0.67±0.43 | 0.78±0.46 |  |  |  |  |  |
| Pelvic balance (cm) | SCT | 0.32±0.39 | 0.39±0.35 | 0.39±0.42 | 4.070, 0.032* | 4.683, 0.038* | 2.211 | 0.131 | None |
|  | CT | 0.40±0.43 | 0.55±0.35 | 0.80±0.48 |  |  |  |  |  |
| Coronal balance (cm) | SCT | 1.08±0.90 | 1.01±0.87 | 0.92±0.67 | 0.549, 0.582 | 0.448, 0.508 | 0.403 | 0.607 | None |
|  | CT | 0.90±0.63 | 0.75±0.65 | 0.91±0.67 |  |  |  |  |  |

Note: T1: Discharge; T2: 3-month postoperative; T3: 6-month postoperative; *: *P*<0.05; **: *P* < 0.01

**TABLE 3** Comparative analysis of convex/concave EMG RMS outcomes and trunk muscular strength and endurance in paraspinal muscles between the two patient cohorts across three testing maneuvers.

| Outcomes | | |  |  |  | Time*Group | | Pairwise  comparison |
| --- | --- | --- | --- | --- | --- | --- | --- | --- |
|  |  |  | T0  Mean ± SD | T2  Mean ± SD | T3  Mean ± SD | F | P |  |
| Static standing  Convex /  Concave RMS | UEV | SCT | 1.33 ± 0.31 | 1.20 ± 0.33 | 1.15 ± 0.41 | 0.409 | 0.648 | None |
|  |  | CT | 1.25 ± 0.34 | 1.19 ± 0.26 | 1.18 ± 0.34 |  |  |  |
|  | AV | SCT | 1.78 ± 0.53 | 1.20 ± 0.26 | 1.19 ± 0.31 | 0.058 | 0.944 | None |
|  |  | CT | 1.61 ± 0.49 | 1.18 ± 0.34 | 1.20 ± 0.35 |  |  |  |
|  | LEV | SCT | 1.48 ± 0.40 | 1.15 ± 0.28 | 1.13 ± 0.39 | 0.656 | 0.503 | None |
|  |  | CT | 1.35 ± 0.32 | 1.18 ± 0.16 | 1.14 ± 0.29 |  |  |  |
| Flexor testing  Convex /  Concave RMS | UEV | SCT | 1.48 ± 0.39 | 1.13 ± 0.27 | 1.21 ± 0.33 | 0.508 | 0.587 | None |
|  |  | CT | 1.33 ± 0.32 | 1.12 ± 0.17 | 1.14 ± 0.20 |  |  |  |
|  | AV | SCT | 1.56 ± 0.73 | 1.17 ± 0.21 | 1.21 ± 0.25 | 0.062 | 0.940 | None |
|  |  | CT | 1.44 ± 0.84 | 1.18 ± 0.22 | 1.17 ± 0.31 |  |  |  |
|  | LEV | SCT | 1.14 ± 0.34 | 1.18 ± 0.21 | 1.11 ± 0.27 | 0.469 | 0.628 | None |
|  |  | CT | 1.26 ± 0.26 | 1.19 ± 0.35 | 1.11 ± 0.19 |  |  |  |
| Extensor testing  Convex /  Concave RMS | UEV | SCT | 1.39 ± 0.73 | 1.21 ± 0.36 | 1.14 ± 0.26 | 0.022 | 0.979 | None |
|  |  | CT | 1.30 ± 0.49 | 1.12 ± 0.32 | 1.09 ± 0.26 |  |  |  |
|  | AV | SCT | 1.54 ± 0.49 | 1.18 ± 0.27 | 1.17 ± 0.43^a^ | 0.252 | 0.778 | None |
|  |  | CT | 1.54 ± 0.70 | 1.25 ± 0.34 | 1.12 ± 0.37 |  |  |  |
|  | LEV | SCT | 1.21 ± 0.35 | 1.13 ± 0.20 | 1.07 ± 0.30 | 0.546 | 0.582 | None |
|  |  | CT | 1.26 ± 0.45 | 1.18 ± 0.22 | 1.14 ± 0.30 |  |  |  |
| Flexor strength | | SCT | 11.91 ± 1.81 | 9.97 ± 2.52 | 11.33 ± 2.36 | 0.633 | 0.529 | None |
|  |  | CT | 11.92 ± 1.85 | 9.77 ± 1.99 | 10.64 ± 2.37 |  |  |  |
| Extensor strength | | SCT | 16.35 ± 3.13 | 13.37 ± 2.56 | 15.16 ± 2.99 | 2.745 | 0.072 | None |
|  |  | CT | 17.39 ± 3.03 | 14.12 ± 2.38 | 14.08 ± 2.49 |  |  |  |
| Flexor endurance | | SCT | 77.83 ± 43.36 | 68.66 ± 36.91 | 83.61 ± 26.22^a^ | 1.428 | 0.247 | None |
|  |  | CT | 76.88 ± 37.35 | 54.66 ± 24.24 | 66.77 ± 22.47 |  |  |  |
| Extensor endurance | | SCT | 112.00 ± 24.46 | 86.50 ± 31.68 | 121.16 ± 41.33^a^ | 18.521 | <0.001 | T0 - T2*  T2 - T3** |
|  |  | CT | 125.72 ± 53.44 | 83.05 ± 23.24 | 93.77 ± 33.08 | 11.869 | <0.001 | T0-T2**  T0-T3** |

Note: T0: preoperative; T2: 3-month postoperative; T3: 6-month postoperative; *: *P*<0.05; **: *P* < 0.01; a: compared with CT, *P*<0.05.

**TABLE 4** Comparative Analysis of SRS-22 Outcomes Between the Two Patient Groups

| Outcomes | |  |  |  |  | Time*Group | | Pairwise  comparison |
| --- | --- | --- | --- | --- | --- | --- | --- | --- |
|  |  | T0  Mean ± SD | T1  Mean ± SD | T2  Mean ± SD | T3  Mean ± SD | F | P |  |
| Pain | SCT | 4.41 ± 0.60 | 3.88 ± 0.65 | 4.31 ± 0.52 | 4.40 ± 0.50 | 0.795 | 0.481 | None |
|  | CT | 4.37 ± 0.52 | 3.98 ± 0.60 | 4.14 ± 0.54 | 4.28 ± 0.48 |  |  |  |
| Function | SCT | 4.27 ± 0.80 | 3.38 ± 0.84 | 3.94 ± 0.48 | 4.23 ± 0.50 | 0.948 | 0.421 | None |
|  | CT | 4.36 ± 0.47 | 3.60 ± 0.66 | 3.91 ± 0.49 | 4.05 ± 0.48 |  |  |  |
| Self-image | SCT | 3.35 ± 0.82 | 3.65 ± 0.46 | 4.02 ± 0.49 | 4.24 ± 0.49^a^ | 8.902 | <0.001 | T0 - T2**  T0 - T3**  T1 - T3** |
|  | CT | 3.57 ± 0.45 | 3.67 ± 0.61 | 3.87 ± 0.58 | 3.77 ± 0.79 | 1.704 | 0.186 | None |
| Mental Health | SCT | 3.91 ± 0.47 | 3.78 ± 0.47 | 3.91 ± 0.59 | 4.08 ± 0.66 | 0.672 | 0.545 | None |
|  | CT | 4.05 ± 0.48 | 3.92 ± 0.61 | 4.00 ± 0.64 | 4.03 ± 0.77 |  |  |  |

Note: T0: preoperative; T1: discharge; T2: 3-month postoperative; T3: 6-month postoperative; *: *P*<0.05; **: *P* < 0.01; a: compared with CT, *P*<0.05.

**Reviewer #3:**

**This study interested me because, based on my clinical experience, minor lumbar curvature can worsen and frontal balance may be affected after thoracic posterior spinal fusion (PSF) surgery, potentially necessitating secondary surgery.
Schroth-based exercises (PSSE), possibly combined with a Chêneau brace, may help prevent the need for additional lumbar fusion.**

**Comment 1：While the study's design and outcome measures were well presented, I hoped for more focus on lumbar curve changes. A longer follow-up might have provided better insights, as no significant changes were reported after six months.**

**Response:**

We sincerely thank the reviewers for their insightful perspectives and constructive comments on our study. The viewpoints raised, grounded in extensive clinical experience, have significantly enhanced our understanding of the potential clinical implications of this research and provided invaluable guidance for refining the discussion section of our manuscript. We have revised the manuscript accordingly based on your suggestions.

We thank the reviewer for this insightful comment. One of primary objectives of our study was indeed to investigate whether targeted Schroth exercises for the non-fused lumbar curve could prevent its compensatory progression following selective thoracic fusion. We acknowledge that the 6-month follow-up is relatively short-term, and as noted, we did not observe significant inter-group differences at this point. We speculate that this may be attributed to our study design, wherein both groups received identical core training for the first 3 months, with the specific Schroth intervention for the treatment group only commencing between 3-6 months. This finding, however, does not contradict the reviewer's point regarding long-term observation. The progression of compensatory lumbar curves is typically a slow process, and clinically meaningful changes may only become apparent over a longer period (e.g., 1-2 years). Consequently, our short-term results should be interpreted as indicating a potential trend for Schroth exercises in maintaining lumbar alignment in the early postoperative phase. Its long-term efficacy in preventing curve progression and the potential need for secondary surgery requires validation through studies with extended follow-up. Future research should prioritize long-term lumbar compensatory changes as a primary focus. We also added this to the discussion（lines 424-434）.

Therefore, we have explicitly acknowledged in the Discussion and Limitations sections that the 6-month follow-up period is a study constraint and could be the reason for the absence of significant differences in the secondary curve. Nevertheless, we observed promising signals in several other meaningful endpoints that did show significant differences at 6 months, particularly in radiographic pelvic balance and trunk extensor endurance. These parameters are critically important for postural balance and functional recovery in postoperative AIS patients, underscoring the potential value of our intervention and the merit of longer-term investigation.

**Comment 2：I believe assessing extensor muscle endurance is crucial for evaluating patient function, while measuring flexor strength may be less important given the potential for paraspinal muscle damage post-PSF.**

**Response：**

We are grateful to the reviewer for underscoring the clinical relevance of extensor endurance. To complement this view, our rationale for evaluating flexor strength and endurance was grounded in the concept of the trunk as a balanced cylinder. The flexors are a fundamental part of this core structure, and their function is theorized to contribute to dynamic trunk stability and balance. Thus, we selected trunk flexion as a secondary endpoint to allow for a more holistic assessment of core muscle function postoperatively. We also added this to the discussion（Lines 486-489).

**Comment 3：Additionally, targeting the right Iliopsoas muscle with specific Schroth exercises could effectively correct left lumbar curvature.**

**Response：**

We thank the reviewer for this insightful comment. They have accurately identified a key biomechanical strategy in our intervention. Indeed, for the lumbar curve, we specifically employed the strategy of targeting the iliopsoas muscle on the concave side. The activation of the concave iliopsoas can generate a corrective force for the lumbar curve and contribute to the restoration of lumbar lordosis. This is further enhanced by the Schroth "5th Pelvic Correction" maneuver, which involves elevating the iliac crest on the convex side and depressing it on the concave side, thereby inducing a corrective tilt to the pelvis. We sincerely appreciate this suggestion and have incorporated a detailed explanation of this specific biomechanical mechanism into the Discussion section of our manuscript. We also added this to the discussion（Lines 443-446）.
